# Deep Western Boundary Current in the South China Sea

**DOI:** 10.1038/s41598-017-09436-2

**Published:** 2017-08-24

**Authors:** Chun Zhou, Wei Zhao, Jiwei Tian, Xiaolong Zhao, Yuchao Zhu, Qingxuan Yang, Tangdong Qu

**Affiliations:** 1 Physical Oceanography Laboratory, Ocean University of China, 238 Songling Road, Qingdao, 266100 P.R. China; 2 Qingdao National Laboratory for Marine Science and Technology, 1 Wenhai Road, Qingdao, 266200 P.R. China; 3 Qingdao Collaborative Innovation Center of Marine Science and Technology, Ocean University of China, 238 Songling Road, Qingdao, 266100 P.R. China; 4 North China Sea Marine Forecasting Center, State Oceanic Administration, 27 Yunling Road, Qingdao, 266061 P.R. China; 5 First Institute of Oceanography, State Oceanic Administration, 6 Xianxialing Road, Qingdao, 266061 P.R. China; 6 Joint Institute for Regional Earth System Science and Engineering, University of California, Los Angeles, CA 90095 USA

## Abstract

Deep western boundary current (DWBC) was observed for the first time by an array of 6 current meter moorings southeast of the Zhongsha Islands in the South China Sea (SCS) deep basin during the period from August 2012 to January 2014. In the mean, the DWBC in the SCS flows southwestward with core velocity of 2.0 cm/s and a volume transport of 1.65 Sv (1 Sv = 1 × 10^6^ m^3^/s). Its temporal variability is dominated by intraseasonal fluctuations with period around 90 days. The main axis of the DWBC, characterized by a low temperature core, tends not to shift with the 90-day fluctuation.

## Introduction

The South China Sea (SCS) is the largest marginal sea in the northwestern Pacific, with its central basin covering more than 1 × 10^6^ km^2^ below 2000 m and a maximum water depth over 5000 m. The only deep connection between the SCS and its ambient oceans is the deep Luzon Strait, through which the North Pacific Deep Water (NPDW^1^) penetrates into the SCS^2–8^, driving a basin-scale cyclonic circulation in the deep SCS^3, 9–14^. As a result of enhanced diapyncal mixing (~10^−3^ m^2^/s) in the deep SCS^15, 16^, the NPDW upwells and finally exits the SCS in the upper and intermediate layers^4, 17–21^. This three dimensional circulation constitutes the South China Sea Throughflow, serving as a heat and freshwater conveyor that is believed to be climatologically important both regionally and globally^20, 22–24^.

**Figure 1:**
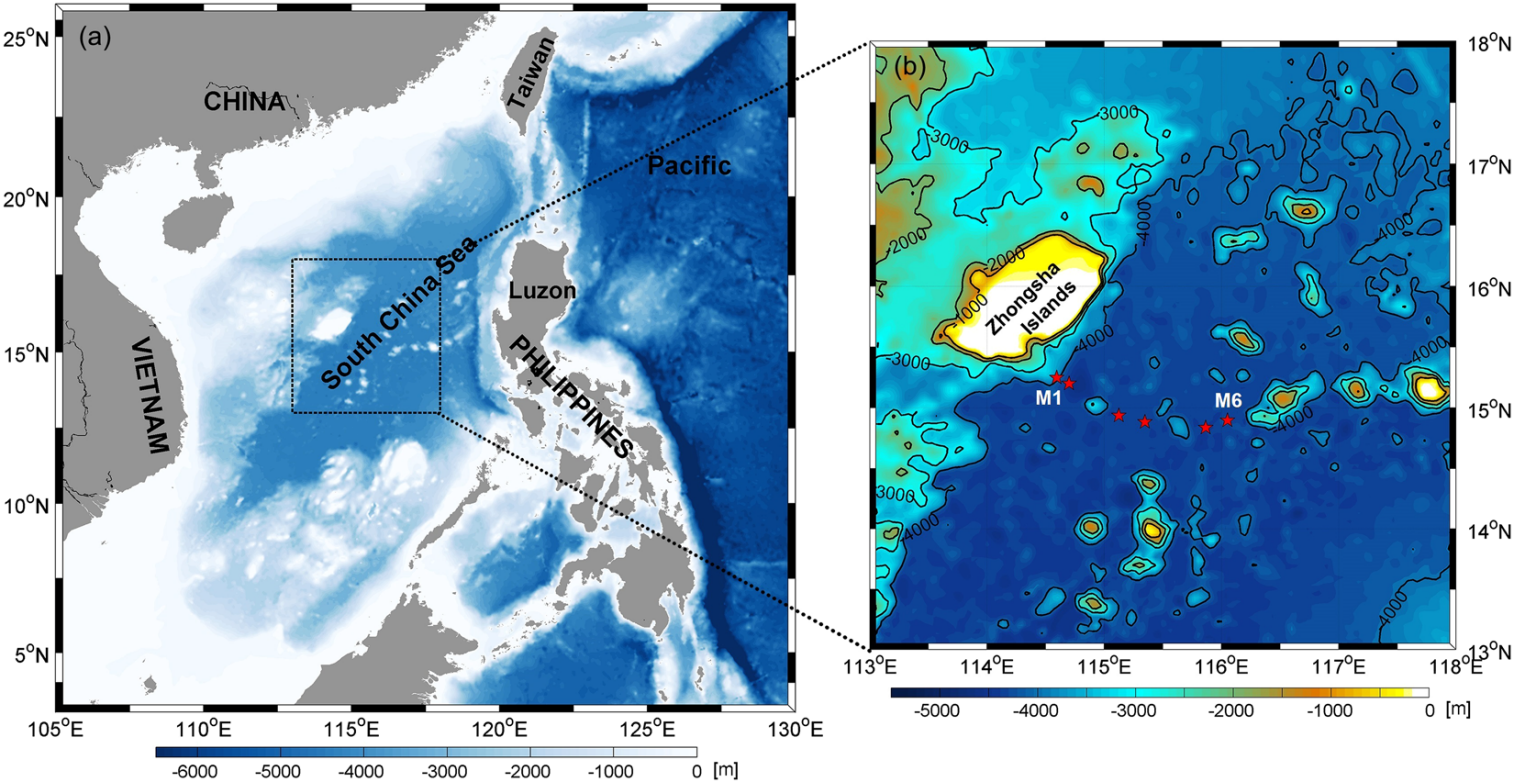
Topography^39^ of the SCS and locations of the moorings. (**a**) Topography of the SCS; (**b**) regional bathymetry indicated by dashed rectangular in the left panel. Mooring locations are shown in red pentagrams in panel (b). Bathymetry data are downloaded from http://topex.ucsd.edu/marine_topo/. Figures are plotted using MATLAB R2013a (http://www.mathworks.com/) with M_Map (a mapping package, http://www.eos.ubc.ca/~rich/map.html).

As NPDW continues to penetrate into the deep SCS, an intensified deep western boundary current (DWBC) is expected to occur^25^, as have been observed in many other deep basins^26–31^. Indeed, previous studies have suggested its existence in the SCS based on geostrophic calculations and numerical simulations^3, 9–14^. Synoptic analysis of a monthly climatology of temperature and salinity from the U.S. Navy Generalized Digital Environment Model indicated the volume transport of the DWBC was 2.92 Sv, with its maximum velocity of 2.7 cm/s. Nevertheless, the existence and characteristics of the DWBC has never been confirmed by direct measurements. Accordingly, an array of six current meter moorings was designed and deployed off the eastern slope of the Zhongsha Islands (Fig. 1) during August 2012-January 2014. This study analyzes the results from these mooring observations and reports the first direct measurements of the DWBC in the SCS.

## Results

### Mean State

We begin with a brief description of the tidal signals, which are ubiquitous and usually not negligible in an abyssal dynamic frame. Butterworth band-pass filtering shows that tidal amplitudes of the U and V components are nearly identical and generally depth-independent. Besides, the diurnal tides dominate over the semi-diurnal tides notably, with their amplitudes reaching ~1.5 and ~1.0 cm/s, respectively. These amplitudes are comparable with the mean value of the current, but are considerably smaller than those of the sub-inertial variability, as will be shown below.

**Figure 2:**
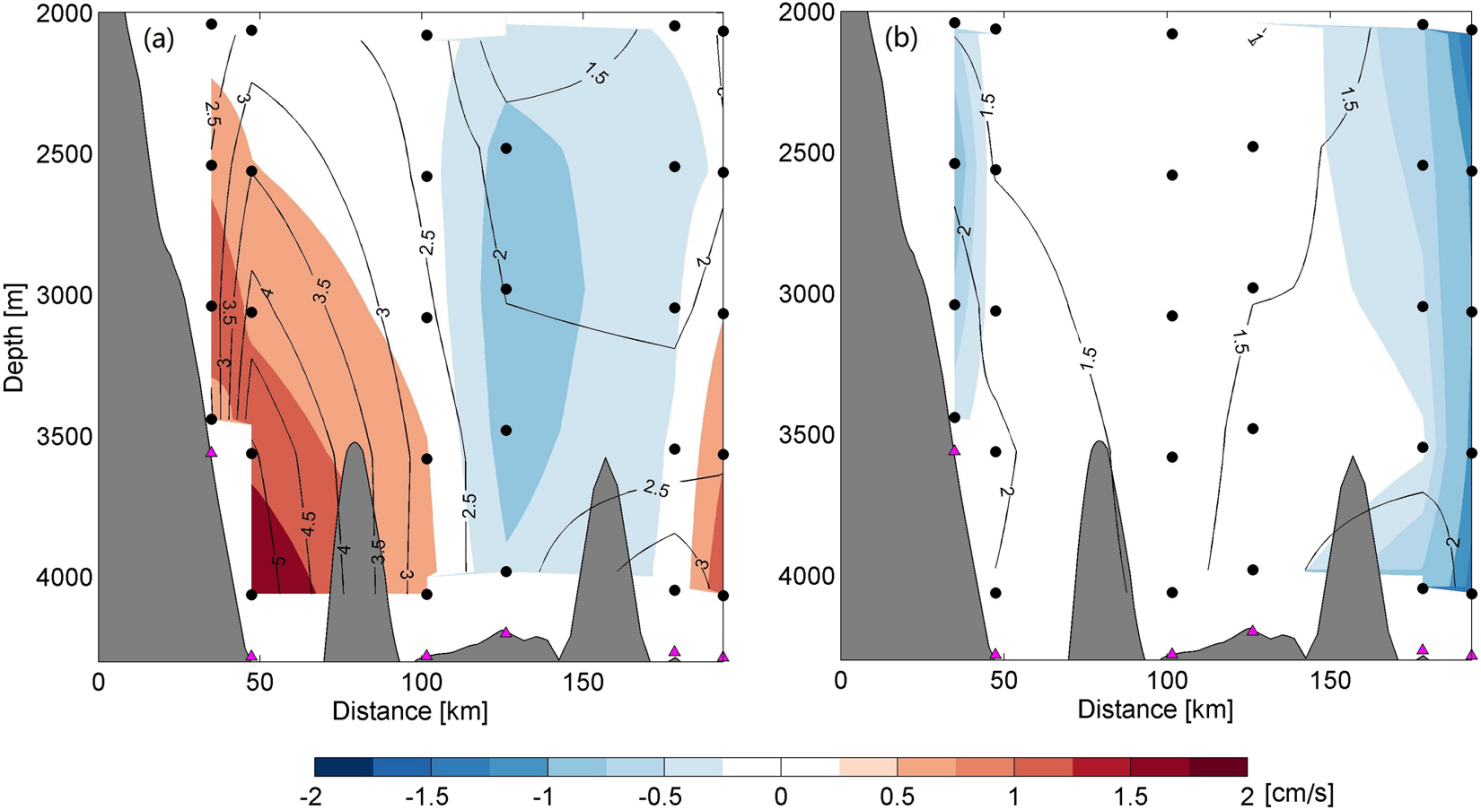
Section view of mean v′ (**a**) and u′ (**b**). Color shading indicates the mean velocity. Gray shading indicates the topography. Black line stands for the standard deviation of the velocity. Mooring locations are indicated in magenta triangles. Locations of current meters are indicated by black dots. Bathymetry data are downloaded from http://topex.ucsd.edu/marine_topo/. Figures are plotted using MATLAB R2013a (http://www.mathworks.com/).

Considering that the DWBC generally follows the topography, we re-coordinate the current into the cross-section (*v*′, generally along the isobaths with positive direction pointing to the southwest) and along-section component (*u*′, perpendicular to *v*′ with positive pointing offshore to the southeast). Observations at M5 and M6 are projected to the section (M1~4). By averaging the daily mean time series over the whole period, Fig. 2 provides a section view of the mean current in the deep western boundary of the SCS. A bottom intensified current is identified right above the bottom of M2 pointing to the southwest, with mean core velocity being ~2.0 cm/s, confirming the existence of the DWBC proposed by previous studies^3, 9–14^. The maximum of mean velocity of the DWBC is slightly weaker than the geostrophic velocity (2.7 cm/s) reported previously^9^. And instead of flowing southward along 116°E as indicated before^9^, the DWBC tends to follow the western boundary and go southwestward of the deep SCS basin. As expected, the DWBC weakens upward, with its upper interface lying at around 2000 m. Horizontally, with its core leaning on the western boundary, the DWBC reaches out for ~100 km offshore. To the east, a weak recirculation is found covering the whole deep layer below 2000 m. This recirculation may result from the topographic effects of the deep seamounts between M4 and M5.

By mapping the mean velocity section of *v*′ on a fine mesh according to the bathymetry, the volume transport of the DWBC below 2000 m is estimated to be 1.65 Sv, with uncertainty of ~0.48 Sv resulting from different extrapolation to the western boundary and bottom. As indicated by previous studies, deep water in the SCS is supplied by the NPDW through the Luzon Strait. Recent results from mooring observation suggest that the volume transport of deep water overflow in the Luzon Strait is around 0.78~0.88 Sv^7, 8^. Considering that the deep water from the Luzon Strait flows directly to the western boundary of the SCS following the topography as shown by previous studies^9, 11^, it implies that the DWBC is not merely supplied by the deep water from the Luzon Strait, but also by water from the interior ocean due to enhanced diapycnal mixing in the northeastern SCS^15, 16^.

### Variability

Numerous previous studies have shown that the DWBC in the ocean varies on various time scales. In the north Atlantic, for example, intraseasonal variability with period ranging from 10 to 60 days has been revealed^32–34^, while in the Southern Pacific the DWBC is modulated by variability with period of 10~50 days^31^.

**Figure 3:**
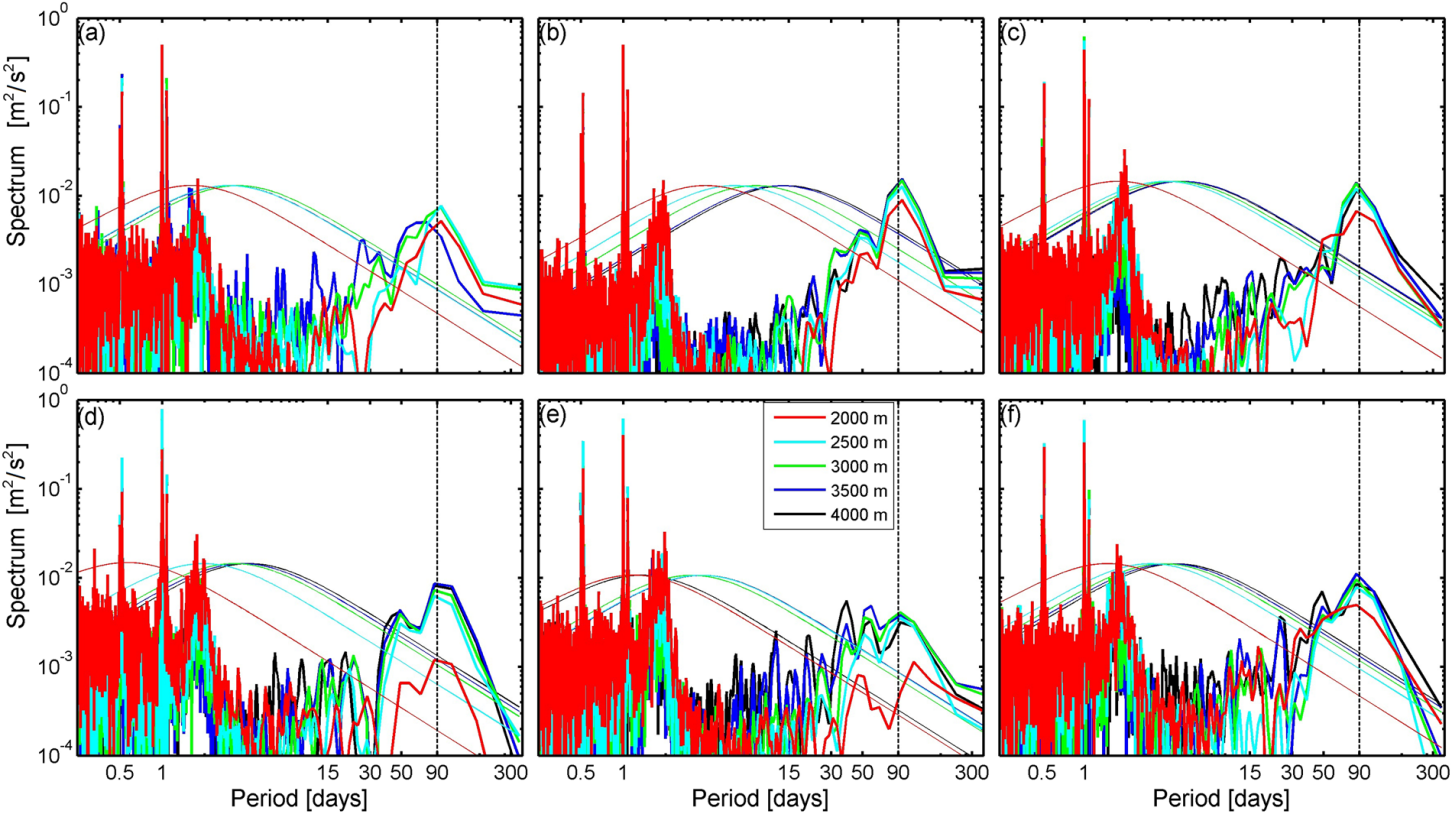
Spectrum analysis of the v′ time series at M1~6 (panel a~f). The bold lines show the power spectra of the v′ time series at different depths indicated in panel (e). The thin lines show the 95% confidence level. Figures are plotted using MATLAB R2013a (http://www.mathworks.com/).

In the SCS, the standard deviations of the DWBC from all current meter observations are considerably larger than its mean values (Fig. 2). In the core region of the DWBC, the standard deviation (5.4 cm/s) of the current is over twice of its mean value, suggesting energetic variability. With this maximum standard deviation coinciding with the DWBC core and decaying toward the upper layer, the standard deviation of the current manifests similar vertical structure to its mean value. According to the daily mean time series of the measurements (see Supplementary Fig. S1), the *v*′ fluctuates between −7.3~20.3 cm/s with its variability generally locating in the intraseasonal band. Spectrum analysis of the original *v*′ shows several peaks in the frequency domain (Fig. 3). While tidal and near-inertial fluctuations dominate the high-frequency variability, the subinertial variability of current is dominated by the 90-day fluctuation at all the mooring sites. To study the characteristics of the 90-day fluctuation, a Butterworth band-pass filter with time window of 70~110 days is applied to all the daily mean *v*′ time series. Vertically, the 90-day fluctuation exhibits coherent phase from 2000 m down to the bottom and to some extent shows a bottom intensification (see Supplementary Fig. S2) similar to that shown in the mean current in Fig. 2a. At M2, for example, the standard deviation of the 90-day fluctuation increases from 2.4 cm/s at 2000 m to 4.9 cm/s near the bottom (around 4000 m). In general, the 90-day fluctuation accounts for ~91% of the total variance in the daily mean time series.

**Figure 4:**
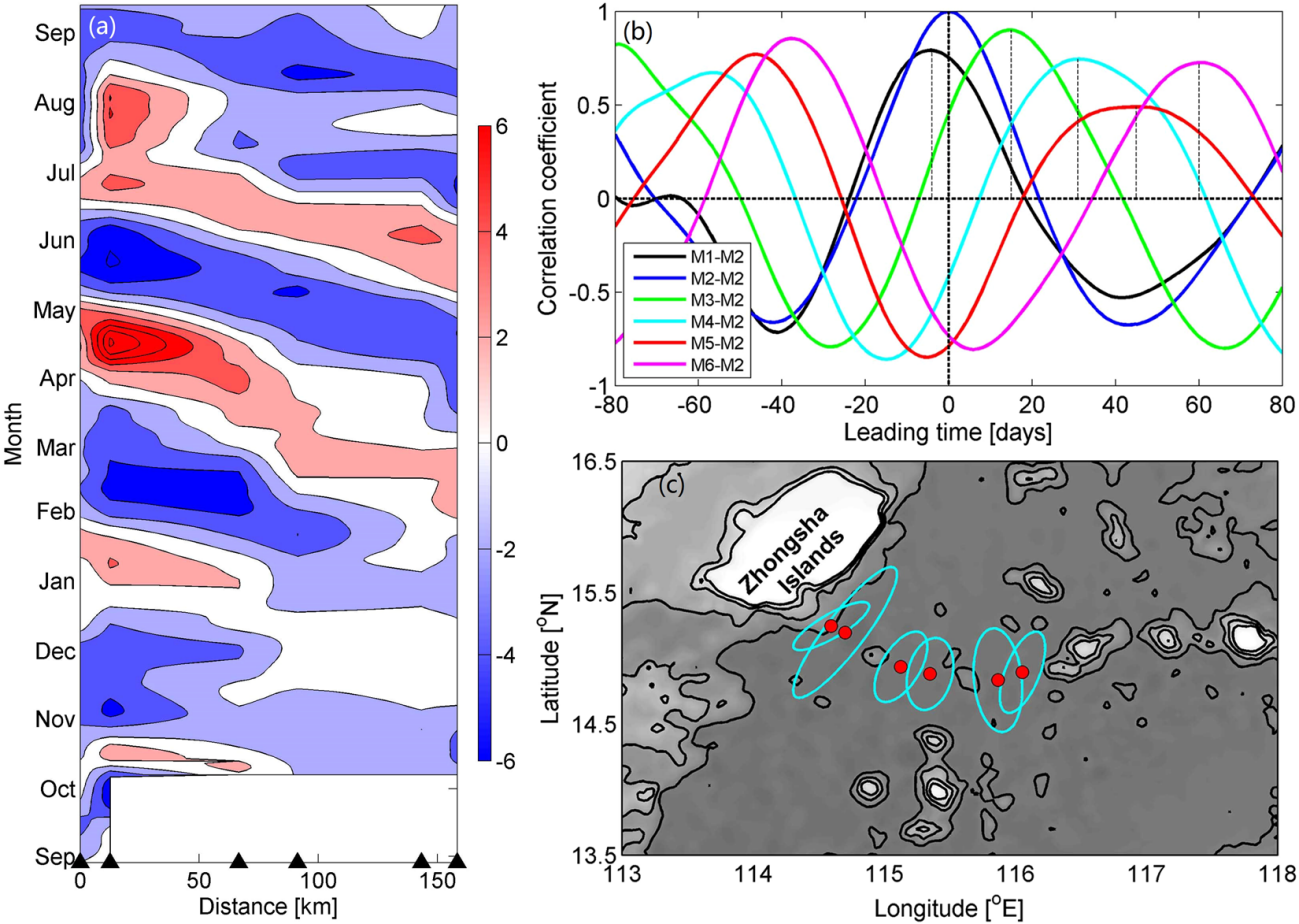
Westward propagation of the 90-day fluctuation. (**a**) Hovmöller diagram of the band-passed v′ at depth of 3500 m; (**b**) Lag correlation coefficient of band-passed v′ between M2 and M1~6 at depth of 3500 m; (**c**) Principle axis variance ellipse of Band-passed velocity at depth of 3500 m. Bathymetry data are downloaded from http://topex.ucsd.edu/marine_topo/. Figures are plotted using MATLAB R2013a (http://www.mathworks.com/).

However, horizontally, 90-day fluctuation is out of phase at different moorings. Hovmöller diagram of band-passed *v*′ is presented to display the propagation of the 90-day fluctuation. An obvious westward propagation is indicated (Fig. 4a). Lag correlation analysis is conducted for the band-passed *v*′ series at 3500 m, where notable lags exist between the western and eastern moorings (Fig. 4b). At Mooring M2, the relative leading time to M1~6 are −4, 0, 14, 32, 47, and 60 days, respectively, corresponding to a mean phase speed of ~2.9 cm/s along the section. Though the propagation direction may be somewhat different with the section, the phase speed of the propagation is quite similar to the typical topographic Rossby wave, a popular candidate for abyssal intraseasonal variability at deep western boundary^35–38^. The elongated ellipses with principal axis roughly following the isobaths (Fig. 4c) are consistent with the characteristics of transverse waves, with principal axis of current variance perpendicular to the phase direction. However, with the western boundary blocked by the Zhongsha Islands, energy of the 90-day fluctuation is expected to radiate from offshore, which requires down-slope phase propagation. This is at odds with the observation here, suggesting that the 90-day fluctuation cannot be attributed to the topographic Rossby wave. Other candidates include the barotropic and baroclinic Rossby waves. We leave this for future studies.

### Hydrographic measurements

After spilling over the deep Luzon Strait, the cold and saline NPDW flows around the northeastern deep SCS basin, and then goes further to the southwest along the western boundary^9, 11^. The core of the DWBC is observed to carry water with mean (standard deviation) potential temperature of 2.086 (±0.003)°C, salinity of 34.619 (±0.001) psu, and consequently potential density (σ_2_) of 36.858 (±0.001) kg/m^3^ (see Supplementary Fig. S3). Comparison with hydrographic properties of the deep water penetrating through the deep Bashi Channel^8^ (1.79 °C and 34.64 psu) suggests that the deep water has experienced notable entrainment and mixing with the relatively warmer and fresher water from the upper layers between the deep Bashi Channel and western boundary of the deep SCS basin. The results presented here are to some extent different with the previous study on the DWBC with potential temperature and salinity of the core being 2.39 °C and 34.625 psu^9^, derived from the GDEM climatology. By linear interpolation, we calculate the potential temperature of the zero-velocity interface of the DWBC to be 2.126 °C.

The 90-day fluctuation could also be identified from the temperature time series. Correlation coefficient between potential temperature and *v*′ at the DWBC core is estimated to be -0.6. But, no substantial correlation is found between salinity and *v*′.

## Discussion

Deep circulation in the SCS has been previously investigated by a couple of studies. Based on density distribution from the World Ocean Database 2001, a cyclonic circulation was suggested^3^. Further analysis of the U.S. Navy Generalized Digital Environment Model (GDEM) climatology showed more detailed structures of the circulation, yielding a mean transport estimate of 3.0 Sv^9^. Despite some quantitative discrepancies, the cyclonic circulation is also simulated by numerical models^10–14^. All these studies indicate the existence of a scientifically significant DWBC in the SCS, which nevertheless has never been confirmed by direct observation. Here, based on current measurements from six year-long current meter moorings, the DWBC of the SCS deep basin southeast of the Zhongsha Islands is observed. The data allows us for the first time to analyze the spatial structure and temporal variability of the DWBC in the SCS. A bottom intensified DWBC with mean core velocity of 2.0 cm/s is identified to flow southwestward along the western boundary below 2000 m. The core of the DWBC sits right above the bottom at about 4000 m, characterized by low temperature and high salinity. The volume transport of the DWBC is 1.65 Sv with uncertainty of ~0.48 Sv. Although somewhat smaller than the previous estimate based on climatological data^9^, our observation is roughly consistent with the deepwater overflow transport through the Luzon Strait^7, 8^.

**Figure 5:**
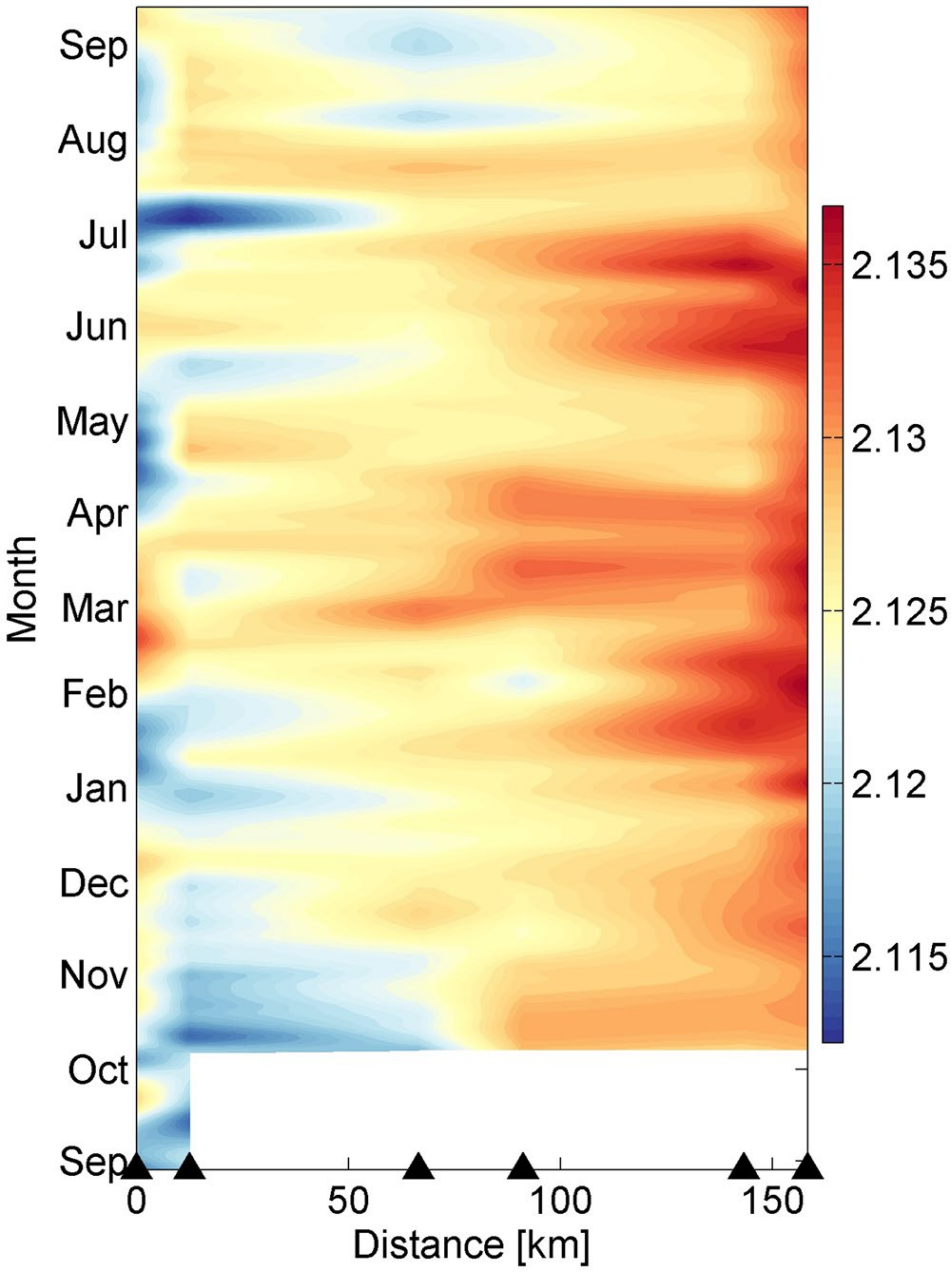
Hovmöller diagram of 15-day lowpassed potential temperature at depth of 3000 m. This figure is plotted using MATLAB R2013a (http://www.mathworks.com/).

Without exception, the amplitude of the DWBC variability exceeds its mean value. Intraseasonal variability with period around 90 days has standard deviation of 4.9 cm/s, which accounts for over 90% of the total variance of the subtidal current. The 90-day fluctuation tends to propagate northwestward with phase speed of ~2.9 cm/s (Fig. 4b), which shifts the core of the southward current westward continuously (Fig. 4a). Inconsistently, the relatively cold water flows across the mooring section at the northwestern end of the section generally throughout the observation period (Fig. 5). As indicated by previous studies, the deep circulation in the SCS is supplied by the cold deep water spilling over the Luzon Strait^9, 11^, implying that the DWBC should be featured by relatively low temperature. Thus, unlike the core of the southward current, the main axis of the DWBC does not shift even with the impact of the 90-day fluctuation.

As an upstream of the SCS deep circulation and the DWBC, the deepwater overflow in the Luzon Strait has been shown to vary on seasonal time scale^7^. How does the DWBC correlate with it? Given that the main axis of the DWBC does not shift notably with time, the daily mean *v*′ time series at about 4000 m in M2 are averaged seasonally to produce the mean seasonal cycle of the DWBC (see Supplementary Fig. S4). The result shows that the DWBC is stronger in spring (March, April, and May) and summer (June, July, and August) than in autumn (September, October, and November) and winter (December, January, and February), with a standard deviation of 2.55 cm/s. With the year-long observation, we are unable to examine the interannual variability and its contribution to the seasonal cycle discussed above. We will leave this for future studies when longer time series become available.

## Methods

### Mooring data

An array of six current meter moorings were positioned at the western boundary of the deep SCS basin (Fig. 1), slightly to the southeast of the Zhongsha Islands, during the period from 28 August 2012 to 11 January 2014. Separated by seamounts, the six moorings were lined roughly perpendicular to the slope. Due to the constraint of cruise arrangements, the moorings were deployed and recovered at different time, with the shortest record of 11 months and the longest record of 15 months. The numbering scheme is such that M1 is the farthest to the northwest, located in the middle of the eastern slope of Zhongsha Islands with water depth of 3500 m, and M6 is the farthest to the east. 29 current meters were mounted on the moorings at nominal depths of 2000 m, 2500 m, 3000 m, 3500 m, and 4000 m (or about 120 m above the bottom), respectively. Aanderaa RCM Seaguard and Nortek Aquadopp (only at the top layers of M3-M6) current meters were employed to equip the moorings. In addition to the Doppler current sensors, the Nortek Aquadopp were assembled with additional temperature and pressure sensors. Besides, the SBE 37-SM Conductivity-Temperature-Depths (CTDs) were mounted on the moorings to measure the hydrographic characteristics of the DWBC. Current meters and CTDs had standard calibration from the manufacturers. The accuracies of the instruments are 0.002 °C for temperature, 0.003 mS/cm for conductivity, 0.1% of full scale range for pressure (which is 7 m for the CTD used in this experiment), and 0.15 cm/s or 1% of reading for current. Details pertinent to the configuration of these moorings are shown in Table 1. All the current meters and CTDs were configured to record data at a sample interval of 1 hour. The instruments yielded satisfactory current and temperature records. The only exception is the CTD on M4 that failed to record data during the second half of the observation.

**Table 1:** Details of mooring configuration.

Mooring ID	Longitude [°E]	Latiitude [°N]	Water depth [m]	Current meter depth [m]				
M1	114°35.761′	15°14.855′	3560	1940	2440	2940*	3440	
M2	114°42.094′	15°11.961′	4282	2062*	2562	3062*	3562	4062*
M3	115°07.607′	14°56.235′	4281	2061#	2561	3061*	3561	4061
M4	115°20.954′	14°52.977′	4200	1980#	2480	2980*	3480	3980
M5	115°51.996′	14°50.133′	4266	2046#	2546	3046*	3546	4046
M6	116°03.241′	14°53.750′	4286	2066#	2566	3066*	3566	4066

*CTD was mounted 5 m below the corresponding current meter. ^#^Current meter assembled with temperature and pressure sensor.

### Estimate of volume transport

To estimate the volume transport of the DWBC across the mooring section, the mean *v*′ at the 29 current meters are linearly extrapolated to the western boundary and to the bottom (1-Minute Gridded topography^39^ is used here). Then the velocity is interpolated and mapped on a finer mesh with horizontal and vertical grid size of 0.1 km and 20 m. Only positive values west of M4, i.e. the southwestward flow, are integrated to calculate the volume transport of the DWBC across the mooring section, which is 1.65 Sv. As the lower limit, the mean *v*′ are linearly interpolated with velocity at the western boundary and the bottom being zero, indicating an estimate of 1.17 Sv. The difference of the volume transport estimated by the two methods, 0.48 Sv, which implies the uncertainty due to the different extrapolations to the western boundary and bottom, is calculated to suggest the main uncertainty of the volume transport.

### Data Availability

Bathymetry data are downloaded from http://topex.ucsd.edu/marine_topo/. The data that support the findings of this study are available from the corresponding author on reasonable request.

## Electronic supplementary material

Supplementary Information
