# Supplementary material for: Deep Western Boundary Current in the South China Sea
Source: Sci Rep. 2017 Aug 24;7:9303. doi: 10.1038/s41598-017-09436-2 (PMC5570915; doi:10.1038/s41598-017-09436-2)
Supplement: Supplementary file 1 — Supplementary Information [file 41598_2017_9436_MOESM1_ESM.doc]

**Supplementary Information**

Deep Western Boundary Current in the South China Sea

Chun Zhou^1, 2, 3^, Wei Zhao^1, 2, 3^*, Jiwei Tian^1, 2, 3^, Xiaolong Zhao^1^, Yuchao Zhu^1^, Qingxuan Yang^1, 2, 3^& Tangdong Qu^4^

1. Physical Oceanography Laboratory, Ocean University of China, 238 Songling Road, Qingdao 266100, P. R. China.
2. Qingdao National Laboratory for Marine Science and Technology, 1 Wenhai Road, Qingdao 266200, P.R. China.
3. Qingdao Collaborative Innovation Center of Marine Science and Technology, Ocean University of China, 238 Songling Road, Qingdao 266100, P. R. China.
4. Joint Institute for Regional Earth System Science and Engineering, University of California, Los Angeles, CA 90095, USA.

*Corresponding author: Wei Zhao (Physical Oceanography Laboratory/CIMST, Ocean University of China and Qingdao National Laboratory for Marine Science and Technology, Qingdao, China; Telephone: +86-532-66786311; Email: weizhao@ouc.edu.cn)


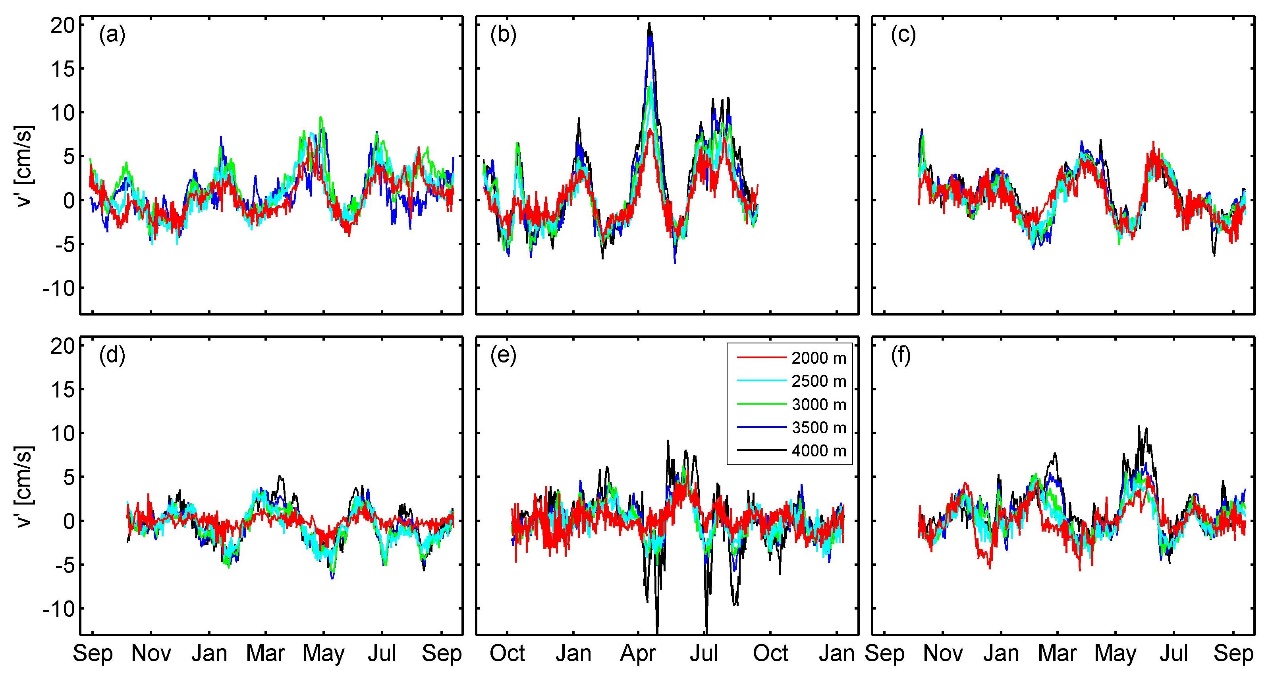


**Figure S1.** Daily mean v’ time series at M1~6 (panel a~f) with different color indicating the observation at different depths. Figures are plotted using MATLAB R2013a (http://www.mathworks.com/).


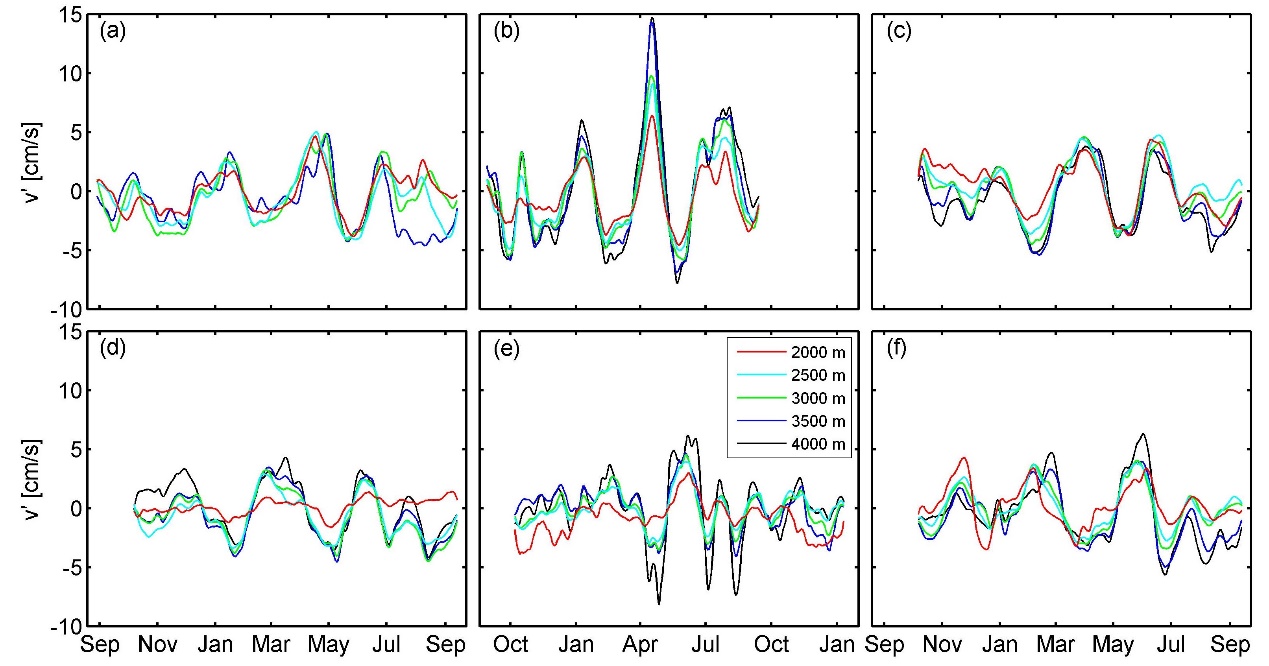


**Figure S2.** Band-passed v’ time series with window of 70~110 days at M1~6 (panel a~f) with different color indicating the observation at different depths. Figures are plotted using MATLAB R2013a (http://www.mathworks.com/).


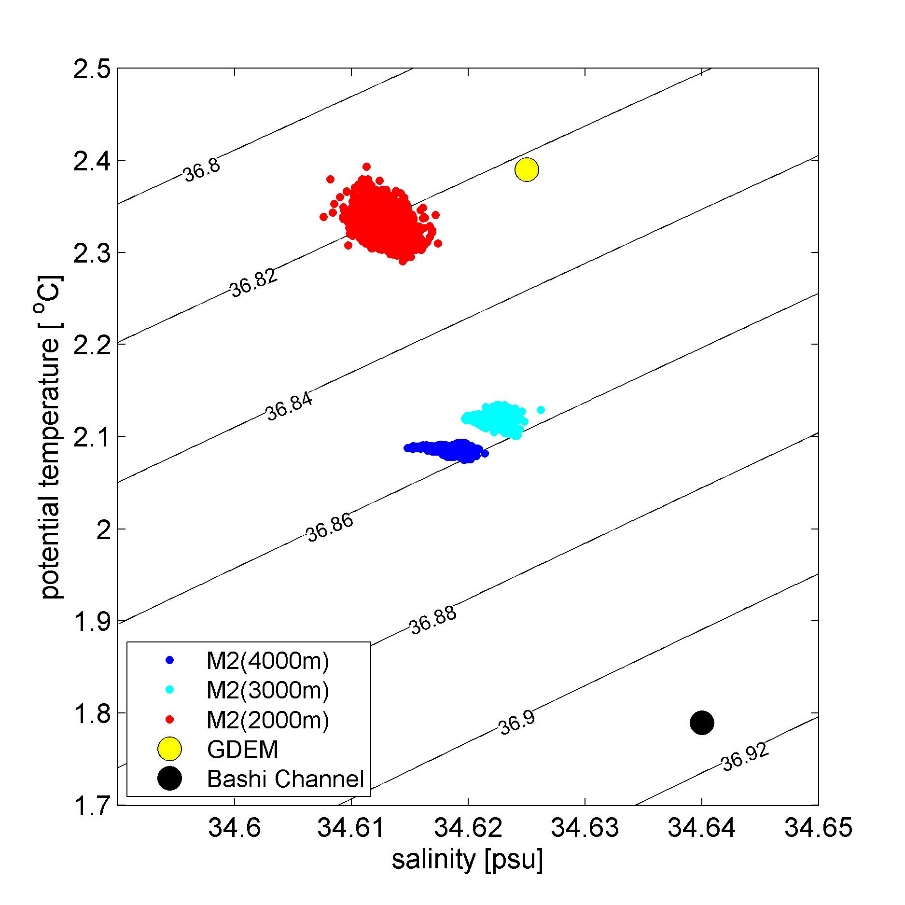


**Figure S3.** Plots of potential temperature - salinity. The blue, cyan, and red dots indicate the CTD observation at M2 at depth of 4000m, 3000m, and 2000m, respectively. The yellow dot stands for the GDEM climatology result at the DWBC (from Wang et al. 2011). The black dot stands for the results of deep water overflow through the Bashi Channel (from Zhao et al. 2016). Black contours show potential density with reference to 2000m depth. This figure is plotted using MATLAB R2013a (http://www.mathworks.com/).


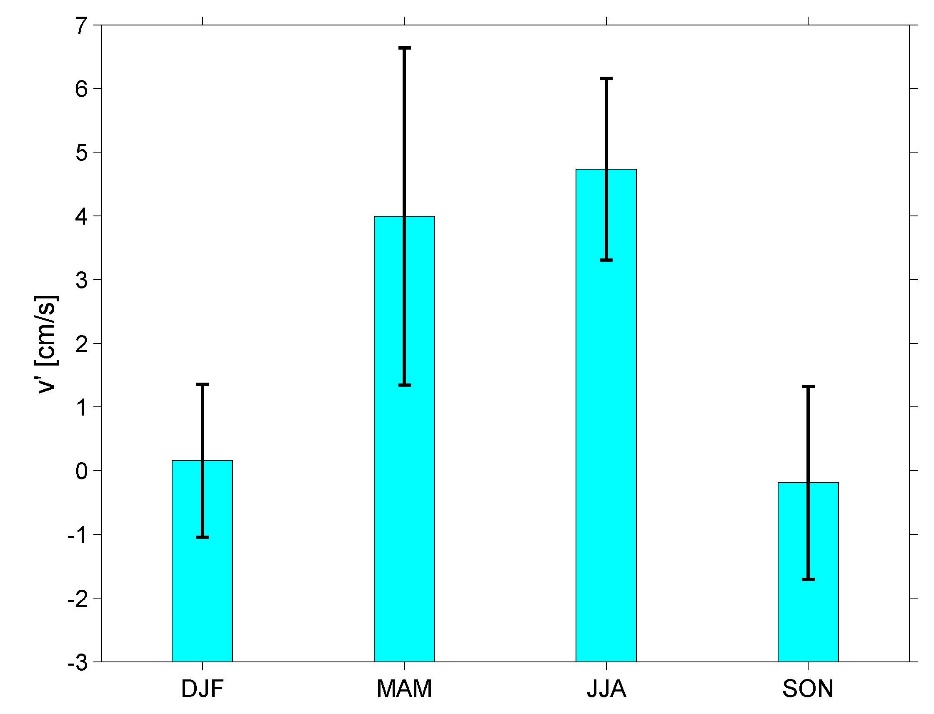


**Figure S4.** Mean seasonal cycle of v’ at M2 based on daily mean v’ time series. Standard deviations are indicated by black bars. This figure is plotted using MATLAB R2013a (http://www.mathworks.com/).
